# Identification of Hydrophobic Interfaces in Protein-Ligand Complexes by Selective Saturation Transfer NMR Spectroscopy

**DOI:** 10.3390/molecules201219824

**Published:** 2015-12-09

**Authors:** Fabien Ferrage, Kaushik Dutta, David Cowburn

**Affiliations:** 1 New York Structural Biology Center, New York, NY 10027, USA; fabien.ferrage@ens.fr (F.F.); kskdutta@gmail.com (K.D.); 2 Department of Chemistry, École Normale Supérieure-PSL Research University, 24 rue Lhomond, 75005 Paris, France; Fabien.ferrage@ens.fr; 3 LBM, Sorbonne Universités, UPMC Univ Paris 06, 4 place Jussieu, F-75005 Paris, France; 4 UMR 7203 LBM, CNRS, F-75005 Paris, France; 5 Department of Biochemistry, Albert Einstein College of Medicine, Bronx, NY 10461, USA

**Keywords:** NMR, cross-saturation, SH3 ligand, interface identification

## Abstract

The proper characterization of protein-ligand interfaces is essential for structural biology, with implications ranging from the fundamental understanding of biological processes to pharmacology. Nuclear magnetic resonance is a powerful technique for such studies. We propose a novel approach to the direct determination of the likely pose of a peptide ligand onto a protein partner, by using frequency-selective cross-saturation with a low stringency isotopic labeling methods. Our method illustrates a complex of the Src homology 3 domain of C-terminal Src kinase with a peptide from the proline-enriched tyrosine phosphatase.

## 1. Introduction

The accurate characterization of protein-protein interfaces is a key element for the understanding of biological mechanisms at a sub molecular level. By providing atomic resolution information of high- or low-affinity complexes, nuclear magnetic resonance (NMR) has proven to be a tool of choice for such studies [1], including development of early leads for therapeutic pharmacology [2,3,4]. The most used NMR method consists in following the chemical shift perturbations on one protein upon titration of the binding partner. This experiment is very sensitive but it provides ambiguous and possibly inaccurate data. Nevertheless, this information can be used to define the interface and dock the structure of a complex [5]. Deficits of this approach include the indirect nature of chemical shift perturbation on complex formation, and ad hoc knowledge-based interpretation. On the other hand, the use of intermolecular Overhauser effects is a more accurate source of information. Filtered Nuclear Overhauser Effect SpectroscopY (NOESY) experiments [6] provide intermolecular distance restraints for structure calculation. In contrast cross-saturation [7,8,9], REDuced/Standard Proton density INTerface identification (REDSPRINT) [10] and the use of enhanced relaxation at the interface [11,12] can provide accurate but partly ambiguous information about the complex. Among all these methods, cross-saturation has the advantage of being both accurate and applicable to large complexes. However, the number of probes is limited, either to nitrogen-bonded or some methyl protons [13,14]. Residue-specific information can reduce the ambiguity of cross-saturation information [15,16] but many samples have to be generated. In all instances, the cost of sample preparation is high due to demanding, specific isotopic labeling schemes. In this paper, we introduce a new selective cross-saturation method that uses many side-chain protons as probes of the interface and relies on a less stringent isotopic labeling scheme.

Saturation-transfer methods are less sensitive to transverse relaxation than experiments based on isotopic filters for instance [10]. Saturation-transfer methods are less sensitive to transverse relaxation in this class of experiments. We demonstrate the benefit from the scarcity of residual protons on the α position in REDPRO samples [17] to carry on selective saturation with little perturbation on either aliphatic of aromatic side-chains. This labeling scheme used [U-^13^C, ^1^H]-d-Glucose as the carbon source is minimal media with D_2_O solvent. This results in a well-defined low density of ^1^H’s and specifically reduces the number of α ^1^H’s because of amino acids synthesis from α-keto precursors incorporating ^2^H from the solvent [17,18] (see Figure S1). Here, this allowed us to carry on selective saturation of a protonated binding partner in the α proton spectral region with significant cross-saturation on aliphatic of aromatic side-chains of the REDPRO labeled protein. The experiment was demonstrated on the complex of Src homology 3 domain (SH3) of C-terminal Src Kinase (CSK) with a reduced proton density [17] and the 25 residue long peptide from the proline-enriched tyrosine phosphatase (PEP) [19].

## 2. Results and Discussion

**Figure 1:**
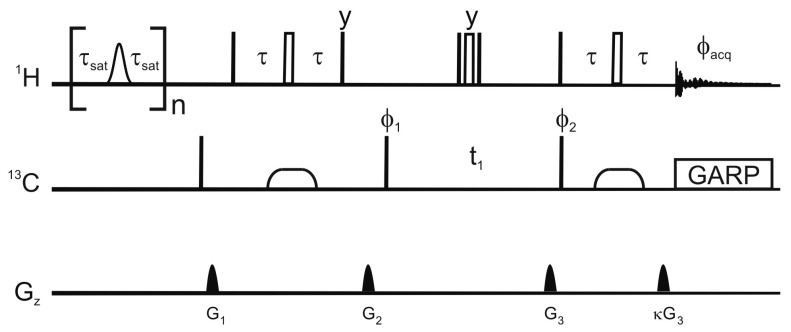
Pulse sequence employed for the selective saturation transfer. The selective pulses employed for saturation are Gaussian shaped π nutations with a 12 ms duration, the delay τ_sat_ is 9 ms, and n is 115. In the reference experiment, the selective pulses are replaced by a 12 ms delay. Narrow and open wide rectangles represent π/2 and π pulses respectively. The phase of a pulse is *x* unless otherwise stated. Broad pulses on the carbon are adiabatic smoothed CHIRP pulses [24], their duration is 500 μs, the sweep 60 kHz and the maximum amplitude 10.4 kHz. The proton radiofrequency carrier is at 4.3 ppm in the saturation scheme and at 4.7 ppm for the HSQC. The carrier for the carbon-13 is at 37.5 ppm or 120 ppm for the detection of aliphatic or aromatic carbons respectively. The delay τ is 1.85 ms or 1.39 ms for the detection of aliphatic and aromatic protons respectively. Decoupling uses a GARP scheme (amplitude 3.8 kHz). The phase cycle is φ_1_ = *x*, −*x*, *x*, −*x*; φ_2_ = *x*, *x*, −*x*, −*x*; and φ_acq_ = *x*, −*x*, −*x*, *x*. The maximum amplitudes and durations of the sine-shaped gradient are G_1_ (8.5 G/cm, 500 μs); G_2_ (35.5 G/cm, 2 ms); G_3_ (40 G/cm, 1 ms); and G_4_ (10.05 G/cm, 1 ms).

Figure 1 shows the pulse sequence employed for this saturation-transfer experiment. The saturation scheme is a series of Gaussian-shaped selective π pulses in the H^α^ region. Detection is carried out with an HSQC scheme [20]. One should notice that the saturation motif is symmetric (*i.e.*, the delay between the last pulse and the observation is twice shorter than the inter-pulse delay). Average Liouvillian Theory [21,22,23] shows that this is critical for fast proton relaxing systems.

**Figure 2:**
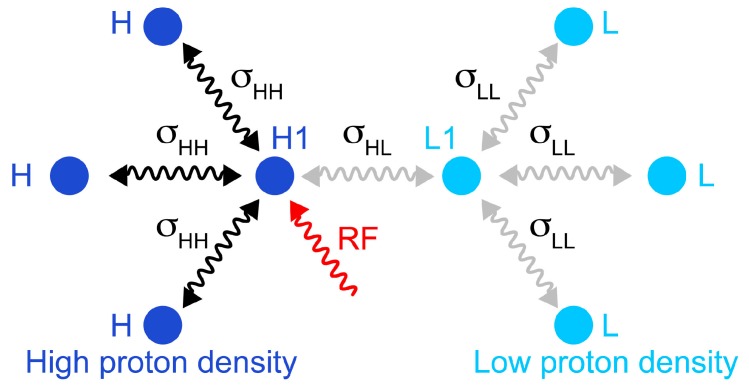
Schematic representation of the simulated spin system, which comprises four protons in a fully protonated protein (**left**) and four protons in a reduced proton density protein (**right**). The cross-relaxation rates within the high proton density are σ_HH_ = 3 s^−1^; cross-relaxation rates involving low proton density sites are σ_HL_ = σ_LL_ = 0.5 s^−1^. Longitudinal relaxation rates are *R*_1_(H1) = 12 s^−1^; *R*_1_(H) = 10 s^−1^; *R*_1_(L1) = 4 s^−1^; *R*_1_(L) = 3.5 s^−1^. The transverse relaxation rate of the irradiated proton was *R*_2_(H1) = 25 s^−1^. The selective saturation was simulated following exactly the parameters presented in Figure 1: Gaussian shaped inversion pulses with a 12 ms duration were applied with a delay τ_sat_ is 9 ms, and *n* was 115.

**Figure 3:**
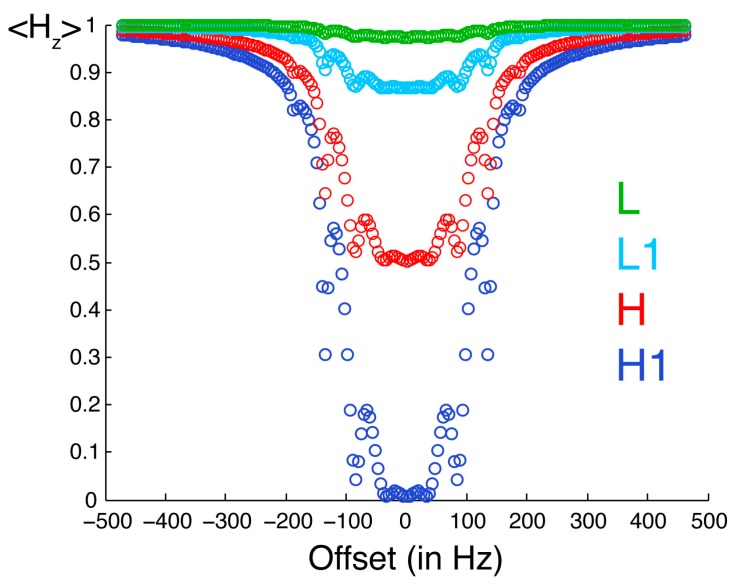
Saturation profiles simulated for the spin system described in Figure 2. The observable longitudinal polarization for proton H1 is shown in dark blue. Equilibrium polarizations are normalized to unity. H1 is the one irradiated by selective radiofrequency pulses. The offset between the resonance frequency of H1 and the carrier frequency of radiofrequency pulses is on the *x*-axis. The longitudinal polarization of protons H, L1 and L are in red, light blue and green, respectively.

Simulations on a model system show that the direct saturation by radiofrequency pulses is very selective. We have simulated a simplified system of 8 protons shown in Figure 2 using the homogeneous master equation [21,25,26]. Figure 3 illustrates the selective saturation profiles for the four types of protons. The irradiated proton is saturated efficiently and selectively. With the parameters employed in our experiments, the efficiency of saturation is about 50% at 0.18 ppm and 10% at 0.31 ppm from resonance, while a residual saturation of 1% is predicted for an offset of 1 ppm on a spectrometer with a ^1^H Larmor frequency of 700 MHz. The neighboring proton L1 is significantly saturated (about 13%). The protons in the low-proton density protein, which are not directly cross-relaxing with the protons in the high proton density protein, are modestly saturated (less than 3%). Thus, saturation is both selective with respect to the chemical shift of the proton irradiated but also selective with respect to the direct proximity of the proton where there is an observed saturation transfer.

**Figure 4:**
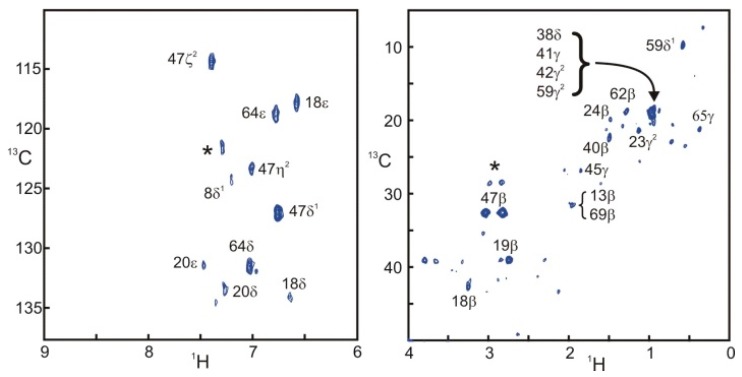
Selective saturation transfer difference ^1^H{^13^C}HSQC spectra of the aromatic and aliphatic side-chains in SH3. Both spectra were obtained with the sequence and parameters presented in Figure 1. Assignments appear directly on the spectra. When several peaks overlap, all assigned resonances appear adjacent to a brace. Stars (*) correspond to unassigned resonances.

**Figure 5:**
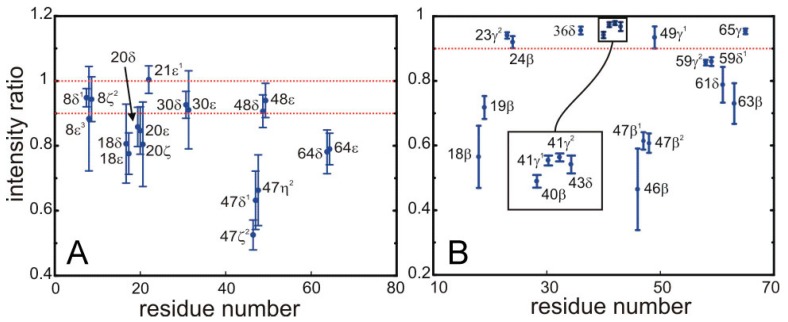
Saturation of the signals from for the ^13^C bonded aromatic (**A**) and aliphatic (**B**) protons of side-chains in SH3 by the sequence employed for the selective saturation transfer. The ratio of intensities in the HSQC with and without the selective saturation scheme is plotted along the *y*-axis. (**A**) The left panel shows the ring aromatics of W8, Y18, F20, H21, F30, W47, Y48, Y64; and (**B**) the right panel the aliphatic protons of the side chains of Y18, N19, T23, A24, L36, A40, V41, K43, N46, W47, I59, P61, N63, V65.

The first step of the data analysis is to compute a saturation transfer difference (STD) spectrum [27]. Carbon-13 labeling permits both the filtering of the signal of the target protein and the recording of two-dimensional spectra dispersed by both ^1^H and ^13^C. Figure 4 presents two STD correlation spectra edited for the aromatic and aliphatic side-chains of SH3. The aromatic spectrum is remarkably similar to the one obtained with filtered NOESY approaches [10]. The aliphatic spectrum shows intense signals for some protons located at the interface. Nevertheless, the computation of the ratio of intensities in the saturated and reference experiments is necessary to separate the effects of sensitivity and saturation. Figure 5 shows this ratio for most of the peaks appearing in the difference spectra displayed in Figure 4. 

The quantitative data displayed in Figure 5 permit the unambiguous identification of protons located at the interface. We have defined a threshold so that only protons displaying more than 10% of saturation are assigned to the interface. Strong saturation effects are observed for some aromatic side-chains, possibly arising from spin diffusion among the relatively close ring protons.

**Figure 6:**
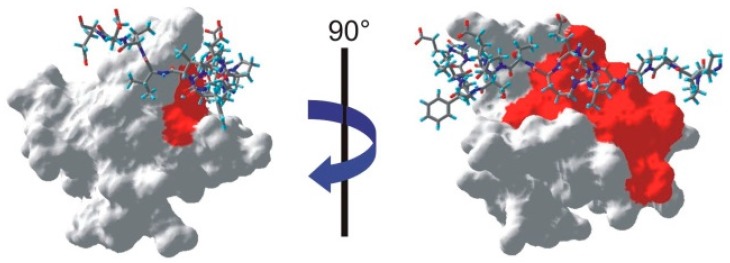
Mapping of the interface on one NMR structure of the SH3-PEP complex (PDB: 1JEG [19]). The peptide appears as a stick-plot lying on the surface of the SH3 domain. Every residue for which one proton is below the threshold shown in Figure 3 appears in red on the surface of the SH3 domain.

All the residues for which at least one proton was identified as saturated are shown on the NMR structure of the SH3-PEP complex [19] in Figure 6. Similarly to our previous study of the complex, we find an extension of the interface on the SH3 domain towards the region facing the characteristic left-handed type II polyproline helix (PPII) of the peptide [10]. Clearly, the interface between the SH3 domain and PEP includes a tight contact between the PPII helix of PEP and the SH3 domain. In addition to confirming this contact between the two partners, we should note that all other residues identified as belonging to the interface are located within 4 Å of the peptide.

However, it is clear from the left hand-side of Figure 6 that a whole part of the interface is not identified by our method. Several factors could contribute to this issue. First, we can exclude the possibility that a large flexibility of this fragment of the peptide on a ns time scale leads to vanishing cross-relaxation terms. Indeed, several studies have identified intermolecular nOe’s in this region of the complex [10,19]. Nevertheless, significant mobility has been identified on both PEP and SH3 in this part of the interface [19]. As a result, lower cross-relaxation rates within the peptide may lead to a less efficient propagation of the saturation. In addition, the available assignment of the peptide proton spectrum show that only one saturated proton in PEP (the Hα of serine 19) is within 3.5 Å of an aromatic proton of the SH3 domain, so that direct saturation transfer should be limited.

Therefore, it is likely that the scarcity of saturation sources on the peptide is at the origin of the insufficient saturation of the SH3 resonances. One may notice that this issue will be less critical in a complex where the binding partner is a larger protein since the extended structure of the peptide in SH3-PEP leads to a poor propagation (in one dimension) of the saturation within PEP. A globular binding partner should show a nearly uniform degree of saturation thanks to the three-dimensional intramolecular propagation of saturation. Alternatively, a series of experiments with variable selective irradiation frequencies could use prior knowledge of chemical shifts distributions in the binding partners. Such a scheme would have several major advantages: (i) the overall efficiency of saturation over the series of experiments would be higher, with a better coverage of the binding interface; (ii) cross-validations between experiments would enhance the accuracy of the overall results; and (iii) statistically significant variations of the saturation transfer between experiments could be correlated to the local distribution of chemical shifts in the source protein to obtain site-specific constraints on both partner so as to guide docking in a manner similar to residue-specific cross saturation [9,15,16]. However, one should be careful with potential artefacts due to direct saturation of the target protein when the saturation frequency is decreased significantly (see Figures S2 and S3).

The small size of the complex studied here offers little indication of the range of sizes of complexes that can be studied with this technique. Figures S2 and S3 show saturation difference spectra obtained on the same complex dissolved in a mixture of D_2_O and perdeuterated Glycerol (21.7% glycerol *w*/*w*). The rotational correlation time for the complex was estimated to be 20 ns, mimicking a 35–40 kDa system [10]. Remarkably, the quality of the spectra is mostly preserved. Admittedly, the small disordered PEP peptide still behaves like a large ligand or small protein so that this experiment only demonstrates the validity of the approach for complexes between a large target protein and a small or disordered peptide. Larger protonated source proteins might require slightly stronger saturation power in order to compensate from fast proton relaxation. In principle, the saturation transfer technique should be applicable in both fast and slow binding exchange regimes. In cases where substantial motion is present in a complex, it may be advantageous to use our selective saturation approach as a confirmation of those contacts which are most occupied—possibly of use in design of new ligands of increased enthalpic and decreased entropic contributions to binding [28,29].

## 3. Experimental Section

The expression and purification of Csk SH3 and the 25 residue-long peptide from the tyrosine phosphatase (PEP) followed the protocol already published [19]. The [^13^C, ^15^N, ^2^H]-Csk SH3 protein was prepared using the REDPRO labeling scheme [17]. The REDPRO labeling scheme mostly consists in growing *E. coli* cells in a deuterated minimal medium supplemented with a protonated source for carbon-13 (here glucose). Such a scheme results in residual random protonation (close to 10%) in an otherwise deuterated background. Importantly for the present study, the α position is almost fully deuterated (see Figure S1). Unlabeled PEP was grown in LB media [19]. The NMR sample of the complex of [^13^C, ^15^N, ^2^H]-Csk SH3 [0.4 mM] and [^1^H]-PEP [1.2 mM] was prepared in 98% ^2^H_2_O buffer (20 mM Tris-d_11_, 150 mM NaCl, 0.1% NaN_3_ and pH 7.2).

Resonance assignments obtained on a fully protonated sample of the SH3 domain have been transferred to the REDPRO sample with a systematic shift of proton and carbon-13 resonance frequencies expected from partial deuteration [30]: −0.3 ppm per deuteron in carbon-13 and −0.02 ppm per deuteron for the geminal proton.

All NMR experiments used a 700 MHz Avance NMR spectrometer (Bruker Biospin, Billerica, MA, USA) equipped with a helium-cooled cryogenic probe, with a *z*-axis gradient. Experiments were collected at 298 K. Details of the NMR pulse sequence are given in the caption of Figure 1.

## 4. Conclusions

We have introduced an experimental protocol based on simple isotopic labeling and simple NMR experiments to provide saturation transfer data on a protein-protein complex. This approach samples the density of protons of a binding partner with a broad variety of probes on the observed protein, namely the protons of the side chains of most residues, in a single experiment on a single sample. Owing to its simplicity, we expect this approach to be widely used for the characterization of interfaces in protein-protein complexes.

## Supplementary Materials

Supplementary materials can be accessed at: http://www.mdpi.com/1420-3049/20/12/19824/s1.
