# Supplementary material for: Identification of Hydrophobic Interfaces in Protein-Ligand Complexes by Selective Saturation Transfer NMR Spectroscopy
Source: Molecules. 2015 Dec 9;20(12):21992–9. doi: 10.3390/molecules201219824 (PMC6332028; doi:10.3390/molecules201219824)
Supplement: Supplementary file 1 [file molecules-20-19824-s001.pdf]

Supplementary Materials: Identification of Hydrophobic Interfaces in Protein-Ligand Complexes by Selective Saturation Transfer NMR Spectroscopy

Fabien Ferrage, Kaushik Dutta and David Cowburn

1. Sparse Proton Labeling at Hα Positions in a REDPRO Protein

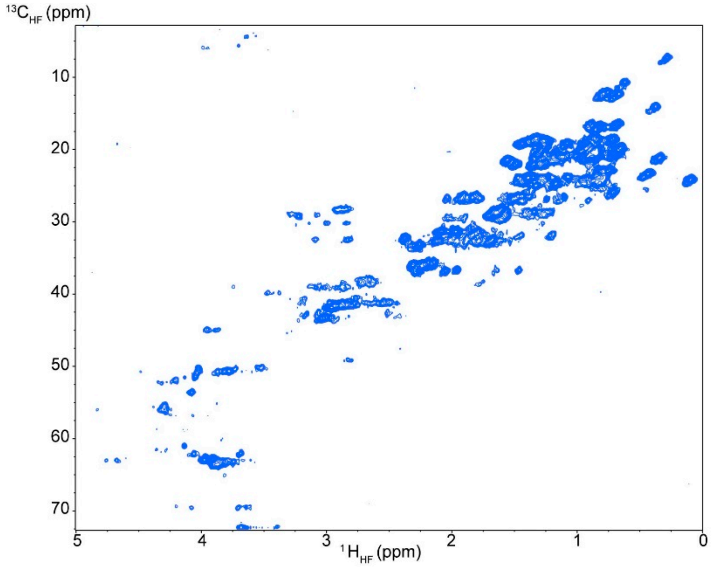

Figure S1. Aliphatic $^1H\{^{13}C\}$ HSQC of the REDPRO SH3 domain bound to unlabeled PEP. The Hα positions are mostly perdeuterated. In particular, the region around 4.5 ppm shows very few resonances. Note that no WATERGATE (or any other selective pulse-based scheme) was used for the suppression of the signal of water.

2. Effect of the Saturation Frequency and Direct Saturation Effects

Selective saturation spectra were recorded on the SH3-PEP system (note that, here, the proteins were dissolved in a mixture of D_2O and Glycerol) with carrier frequencies during saturation at 4.3 ppm, 4.0 ppm and 3.7 ppm. We show in Figures S2 and S3 the difference between the reference and selectively saturated spectra.

The aliphatic HSQC (see Figure S2) displays signs of weak direct saturation effects down to 3.0 ppm when the carrier is placed at 4.0 ppm. In addition, small differences can be observed in the region below 2.5 ppm, with a few small peaks appearing in the difference spectrum. Most importantly, the intense peaks in the difference spectrum are mostly unaltered by the change of carrier frequency.

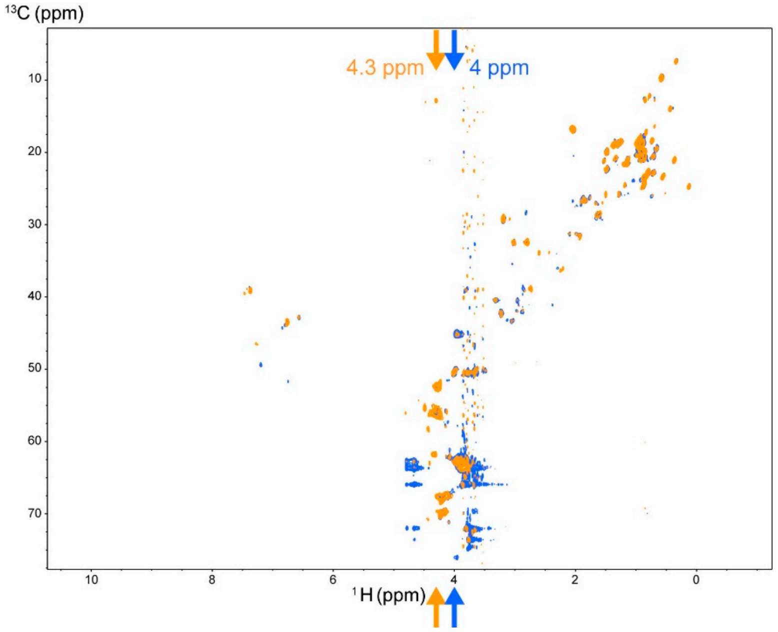

Figure S2. Saturation difference aliphatic $^{1}H\{^{13}C\}$HSQC of the SH3 domain bound to unlabeled PEP with saturation at 4.3 ppm or 4 ppm.

In the aromatic HSQC spectrum (see Figure S3), the overall shape of the spectrum is very similar with saturation at 4.3, 4.0, and 3.7 ppm. However, the intensity of some peaks in the difference spectrum increase significantly when the carrier frequency is decreased (from 4.3 to 3.7 ppm). This can be due to two different contributions. (1) Intramolecular indirect saturation effects could become non negligible, so that residual protons with chemical shifts close to 3.7 ppm in the SH3 domain will lead to some indirect saturation of aromatic protons. Irradiation at 3.7 ppm would be less selective; (2) Alternatively, saturation at 3.7 ppm, closer to most side-chain resonances in PEP, is more efficient to obtain saturation transfer towards aromatic side-chains. Irradiation at 3.7 ppm would be more efficient. Most likely, the observed effect is a combination of the two with saturation at 3.7 ppm both more efficient and less selective.

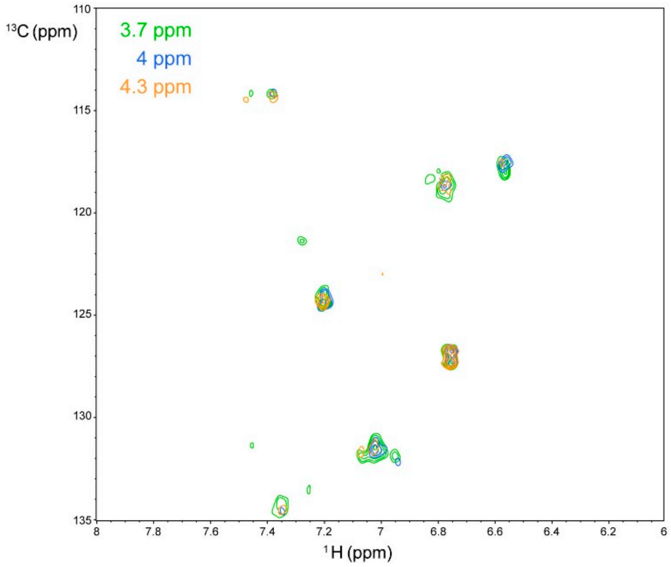

Figure S3. Saturation difference aromatic $^{1}H\{^{13}C\}$HSQC of the SH3 domain bound to unlabeled PEP with saturation at 4.3 ppm, 4 ppm, or 3.7 ppm.
